# Effect of plasma sodium concentration on blood pressure regulators during hemodialysis: a randomized crossover study

**DOI:** 10.1186/s12882-018-0997-z

**Published:** 2018-08-22

**Authors:** Esmée M. Ettema, Johanna Kuipers, Martijn van Faassen, Henk Groen, Arie M. van Roon, Joop D. Lefrandt, Ralf Westerhuis, Ido P. Kema, Harry van Goor, Ron T. Gansevoort, Carlo A. J. M. Gaillard, Casper F. M. Franssen

**Affiliations:** 1 Department of Internal Medicine, Division of Nephrology, University Medical Center Groningen, University of Groningen, PO box 30, 001 9700 RB Groningen, The Netherlands; 2 Dialysis Center Groningen, Groningen, The Netherlands; 3 Department of Laboratory Medicine, University Medical Center Groningen, University of Groningen, Groningen, The Netherlands; 4 Department of Epidemiology, University Medical Center Groningen, University of Groningen, Groningen, The Netherlands; 5 Department of Vascular Medicine, University Medical Center Groningen, University of Groningen, Groningen, The Netherlands; 6 Department of Pathology, University Medical Center Groningen, University of Groningen, Groningen, The Netherlands

**Keywords:** Hemodialysis, Sodium, Endothelium, Sympathetic activity, Vasopressin

## Abstract

**Background:**

Intradialytic hypotension is a common complication of hemodialysis. The Hemocontrol biofeedback system, improving intradialytic hemodynamic stability, is associated with an initial transient increase in plasma sodium levels. Increases in sodium could affect blood pressure regulators.

**Methods:**

We investigated whether Hemocontrol dialysis affects vasopressin and copeptin levels, endothelial function, and sympathetic activity in twenty-nine chronic hemodialysis patients. Each patient underwent one standard hemodialysis and one Hemocontrol hemodialysis. Plasma sodium, osmolality, nitrite and nitrate (NOx), endothelin-1, angiopoietins-1 and 2, and methemoglobin as measures of endothelial function, plasma catecholamines as indices of sympathetic activity and plasma vasopressin and copeptin levels were measured six times during each modality. Blood pressure, heart rate, blood volume, and heart rate variability were repeatedly monitored. Generalized Estimating Equations was used to compare the course of the parameters during the two treatment modalities.

**Results:**

Plasma sodium and osmolality were significantly higher during the first two hours of Hemocontrol hemodialysis. Overall, mean arterial pressure (MAP) was higher during Hemocontrol dialysis. Neither the measures of endothelial function and sympathetic activity nor copeptin levels differed between the two dialysis modalities. In contrast, plasma vasopressin levels were significantly higher during the first half of Hemocontrol dialysis. The intradialytic course of vasopressin was associated with the course of MAP.

**Conclusions:**

A transient intradialytic increase in plasma sodium did not affect indices of endothelial function or sympathetic activity compared with standard hemodialysis, but coincided with higher plasma vasopressin levels. The beneficial effect of higher intradialytic sodium levels on hemodynamic stability might be mediated by vasopressin.

**Trial registration:**

ClinicalTrials.gov. Identifier: NCT03578510. Date of registration: July 5th, 2018. Retrospectively registered.

## Background

Intradialytic hypotension is a serious complication of conventional thrice-weekly hemodialysis treatment, estimated to occur in up to 20–30% of the dialysis treatments. Frequent dialysis hypotension is associated with a lower quality of life and increased cardiovascular morbidity and mortality [1–3].

The dialysate sodium concentration is one of the determinants of hemodynamic stability during hemodialysis, with higher dialysate sodium concentrations resulting in higher intradialytic blood pressures and less dialysis hypotension [4–8]. A common assumption is that the improved hemodynamic stability during dialysis with higher dialysate sodium concentration is based on a higher plasma refill rate as a result of an increase in plasma sodium and thus osmolality [2, 4, 7, 9]. However, various other mechanisms could also play a role. We previously found higher plasma levels of the vasoconstrictor vasopressin during hemodialysis with initially higher dialysate sodium concentrations and, subsequently, higher plasma sodium levels during the first half of hemodialysis [10]. A rise in plasma sodium concentration during hemodialysis could also affect various other blood pressure regulating systems. There is accumulating evidence that increases in plasma sodium concentration may influence vascular endothelial function [11–14] and reduce the release of the vasodilator nitric oxide (NO) by endothelial cells [11, 15–17]. Additionally, an increase in plasma sodium concentration may have a direct stimulating effect on the sympathetic nervous system [18–22].

This study investigated whether the biofeedback system Hemocontrol, characterized by initially higher dialysate and plasma sodium levels as a model for an acute and transient increase of plasma sodium levels, is associated with a different intradialytic course of vasopressin and its surrogate marker copeptin and various markers of endothelial function and sympathetic activity compared with standard hemodialysis.

## Methods

### Patients, design and setting

Prevalent hemodialysis patients from the Dialysis Center Groningen, an independent provider of dialysis services in the northern part of the Netherlands, were prospectively included in this cross-over study between September 2012 and March 2013. Eligibility criteria for inclusion were age ≥ 18 years, a thrice-weekly 4 h hemodialysis schedule, dialysis on an arteriovenous fistula and a usual interdialytic weight gain of ≥2.0 kg. Patients were studied during the first hemodialysis treatment of the week since ultrafiltration volume and blood volume changes are most pronounced after the longest interdialytic interval. Each participating patient underwent one standard hemodialysis and one hemodialysis with Hemocontrol in random order. The Hemocontrol system is designed to prevent large and sudden decreases in blood volume to improve intradialytic hemodynamic stability. The system guides the patients’ blood volume along a predefined ideal relative blood volume trajectory, by continuously adjusting ultrafiltration volume and dialysate conductivity. Changes in blood volume are calculated from changes in hematocrit measured by Hemoscan, a dialysis machine-integrated relative blood volume monitor. The pre-set ideal blood volume curve has a marked decrease in the beginning of the dialysis session, whereas it is more stable during the second half of the treatment [23]. Hallmark of the Hemocontrol system is the combination of a higher ultrafiltration rate and higher dialysate conductivity during the first half of the dialysis session. This results in a more pronounced initial decrease in blood volume and higher plasma sodium levels during the first half of the dialysis session. Since Hemocontrol uses higher ultrafiltration rates during the first half of treatment, lower ultrafiltration rates are used during the second half of the dialysis session, which is considered to be the hemodynamically the most critical part of the treatment. Medication use was similar at both treatments, as well as the posture (half-supine). Patients were asked to refrain from caffeine containing products from midnight prior to both study days. After the first hour of hemodialysis patients received a light meal and coffee or tea. The study was performed in accordance with the principles of the Declaration of Helsinki and the study was approved by the Medical Ethical Committee of the University Medical Center Groningen. All patients gave written informed consent. The study was registered at the CCMO-Register (file number NL39186.042.12 https://www.toetsingonline.nl) prior to enrolment of patients and was retrospectively registered at ClinicalTrials.gov (Identifier: NCT03578510).

### Hemodialysis treatment

Both Hemocontrol and standard hemodialysis were conducted on an Artis hemodialysis machine (Gambro Lundia AB, Lund, Sweden) with a low-flux polysulphon dialyzer F8 or F10 (Fresenius Medical Care, Bad Hamburg, Germany). The ultrafiltration volume was set to achieve dry weight at the completion of the hemodialysis session. Prescriptions regarding dry weight were made by the nephrologists during their weekly visit to the participating patients. Dry weight was evaluated clinically (peripheral edema, signs of pulmonary congestion, intradialytic and interdialytic blood pressure course) in combination with the cardio-thoracic ratio on chest radiography. Blood flow and dialysate flow rates were 300–400 mL/min and 500–700 mL/min, respectively and dialysate temperature was 36.0 or 36.5 °C. These settings were identical for the individual patient at both treatments. Dialysate composition for standard hemodialysis was sodium 139 mmol/L, magnesium 0.5 mmol/L, chloride 109 mmol/L, bicarbonate 34 mmol/L, acetate 3.0 mmol/L and glucose 1.0 g/dL. Dialysate potassium concentration varied between 1 and 3 mmol/L and calcium varied between 1.25 and 1.50 mmol/L depending on the prevailing plasma potassium and calcium concentration. Treatment conditions were identical for the individual patient during both treatments, except the dialysate sodium concentration. Dialysate conductivity was set at 13.9 mS/cm during standard hemodialysis. During Hemocontrol dialysis, the equivalent conductivity was set at 13.9 mS/cm, indicating an identical net sodium removal compared with standard hemodialysis, with lower- and upper tolerance limits of 13.3 and 16.0 mS/cm.

### Outcomes and measurements

The primary outcome variable was intradialytic plasma vasopressin. Additional outcome variables were the intradialytic levels of copeptin, indices of endothelial function and sympathetic activity. Copeptin is a fragment of the vasopressin precursor preprovasopressin and is increasingly used as a surrogate marker of vasopressin [24]. Intradialytic courses of blood pressure, plasma sodium and plasma osmolality were studied during both treatment modalities. Endothelial function was additionally assessed by measurement of endogenous NO production and endothelium-derived endothelin-1 and angiopoietin-1 and 2 (Ang1 and Ang2). Endogenous NO production during hemodialysis was estimated the by measurement of its stable metabolites nitrite and nitrate (NO_2_^−^ and NO_3_^−^, together abbreviated as NOx) and methemoglobin (metHb) [25, 26], since NO itself has a very short half-life [26, 27]. Sympathetic activity was investigated by assessment of heart rate variability (HRV) and baroreflex sensitivity (BRS) using a Finometer and by measurement of the catecholamines dopamine, noradrenalin, and adrenalin. A more detailed description of the Finometer measurements is provided below.

Measurements were performed at the initiation of hemodialysis and thereafter at 30, 60, 120, 180 and 240 min on dialysis. Blood sampling at 240 min of dialysis was performed before blood re-entry to the patient, excluding a hemodilution effect on postdialysis levels of the analytes. All analyses were performed in laboratories of the University Medical Center Groningen, unless stated otherwise. Blood samples for the determination of sodium and osmolality were collected in heparin-coated tubes and measured with the indirect method of ion-selective electrode (Roche Modular, Mannheim, Germany) and by freezing-point depression (Osmo Station Osmometer, Kyoto, Japan), respectively. Blood samples for the determination of hemoglobin and metHb were collected in an electrolyte-compensated heparin coated PICO syringe and analyzed by spectrophometric measurement (ABL 800 Radiometer). Blood samples for the determination of NOx, endothelin-1, Ang1 and Ang2, dopamine, noradrenalin, adrenalin, vasopressin and copeptin were collected in ethylenediaminetetraacetic acid tubes (EDTA) tubes and immediately (within 1 min of blood sampling) centrifuged at 4 °C, at 2500 g for 10 min (Heraeus Biofuge primo R, Hanau, Germany). Next, the samples were stored at − 80 °C until procession. Vasopressin was measured by radio immunoassay (DRG International Inc., Springfield, New Jersey, USA) in the General Clinical Laboratory of the IJsselland Hospital (Capelle a/d IJssel, The Netherlands). Copeptin was measured by an automated sandwich immunoflorescent assay (CT-proAVP; Thermo Fisher Scientific, B.R.A.H.M.S. GmbH, Hennigsdorf, Germany). Dopamine, noradrenalin and adrenalin were analyzed with high-performance liquid chromatography with tandem mass spectrometry, as essentially described elsewhere [28]. Nitrite and nitrate levels were determined using the Griess reaction, as described elsewhere [27]. Ang1 and Ang2 were measured using a solid phase sandwich ELISA (R&D Systems Inc., Minneapolis, Minnesota, USA). Endothelin-1 was measured using a solid phase ELISA with sandwich enzyme immunoassay technique (R&D Systems Inc., Minneapolis, Minnesota, USA).

At the same time-points of blood sampling, the cumulative ultrafiltration volume was registered. Changes in relative blood volume (∆RBV) were calculated from the change in hemoglobin levels as follows: ((Hb_t0_/Hb_t1_)-1) × 100, in which Hb_t0_ and Hb_t1_ represent the hemoglobin levels prior to and during hemodialysis, respectively. The change in RBV normalized for ultrafiltration volume (∆RBV/UF) was calculated as an estimate of plasma refill rate. Blood pressure and heart rate were measured before and directly after the start of dialysis, at 30, 60, 120, 180 min intradialysis, just before the end of dialysis at 240 min and approximately 15 min after dialysis by an automatic oscillometric monitor incorporated in the hemodialysis-apparatus.

### Finometer measurements

To record cardiovascular signals, a non-invasive finger blood pressure device (Finometer, Finapress Medical Systems, Amsterdam, The Netherlands) was used with Beatscope Software (Beatscope Easy version 1.02, Finapres Medical Systems). Systolic blood pressure was derived from beat-to-beat blood pressure measurement, using a finger cuff. Simultaneously, ECG recordings were made using the ECG-module of the Finometer. R-peak detection was performed using dedicated trigger software [29]. Artefact correction and spectral analysis were performed using CARSPAN (version 1.37, University of Groningen, The Netherlands) for the assessment of HRV. This assessment was made with time-domain analysis of the interbeat intervals (NN intervals). The standard deviation, SDNN, is considered a measure of the overall autonomic function [30, 31]. HRV is defined as low when SDNN is < 70 ms. Spectral analysis was used to calculate the low frequency (LF, 0.04–0.15 Hz) and the high frequency (HF, 0.15–0.40 Hz) in the power spectrum of the HRV. To assess whether there is a shift in the autonomic balance toward more sympathetic activity, the LF/HF ratio is used. An increase in the LF/HF ratio indicates more sympathetic activity and/or less parasympathetic activity, whereby HF represents vagal modulation, whereas LF is both vagally and sympathetically modulated. BRS (in ms/mmHg) is considered to be the ratio of the change in heart rate and the change in blood pressure, i.e. the change in interbeat interval related to the simultaneous change in blood pressure [32, 33] The BRS is defined as low when < 3.0 ms/mmHg.

Measurements of HRV and BRS were performed at the initiation of hemodialysis, at 30, 60, 120, 180 min on hemodialysis and at the end of the treatment, for a duration of 10 min for each measurement. When values for HRV or BRS were missing for patients at one treatment modality, the same time point in the other treatment modality was excluded from analysis. HRV and BRS were determined from data segments of minimal 100 up to maximal 300 s.

### Correction for hemoconcentration

Considering the Sieving characteristics of low-flux polysulphone artificial dialyzer and according to the criteria proposed by the Uremic Toxin Work Group, molecules with a molecular weight between 500 and 6000 Da are presumably only partially or not at all removed with hemodialysis [34, 35]. Therefore, plasma levels of vasopressin (≈1000 Da), Ang1 and Ang2 (≈70 kDa), and ET-1 (≈2492 Da) were corrected for hemoconcentration by dividing the measured concentration by a correction factor, Hp (hemoconcentration for the plasma component). This correction factor was calculated using the following formula: Hp = (H_t1_(100-H_t0_))/(H_t0_(100-H_t1_)), were H_t0_ and H_t1_ refer to the concentration before and during hemodialysis, respectively [36].

### Statistical analysis

Assuming a 90% difference in plasma vasopressin levels between standard hemodialysis and Hemocontrol hemodialysis, based on a previous study [10], it was determined that at least 23 patients in a crossover design were needed to achieve 82% power to detect this difference with an alpha of 0.05. Normally distributed variables are represented as mean ± SD or 95% confidence interval (CI), and variables with a skewed distribution are represented as median and interquartile range (IQR). Categorical data are represented as number and percentage. Differences in parameters at baseline and at the end of dialysis and between the two treatment modalities were analyzed with a paired t-test or Wilcoxon Signed Rank test when appropriate. Generalized Estimating Equations (GEE) was used to test whether the intradialytic course of the study parameters differed between treatment modalities, using a fixed correlation between measurements (Exchangeable correlation matrix). When the GEE indicated a significant difference between treatments over time, a post-hoc test (i.e. paired T-test or Wilcoxon signed rank test) was used to compare the individual time points. As sensitivity analysis, all GEE analyses were also performed with correction for age, gender, diabetes mellitus, dialysis vintage, and residual renal function.

Plasma levels of endothelin-1, Ang1 and Ang2, vasopressin and copeptin were corrected for hemoconcentration (as described above) and the corrected values were used in the analyses. As a sensitivity analysis, we also performed the same analyses with the uncorrected values. Additionally, the intradialytic course of systolic and diastolic blood pressure and MAP were also compared between the two treatment modalities using GEE with the measurement directly after the start of dialysis instead of the predialysis measurement. Also, predialysis indices of HRV and BRS were compared between patients using a beta-blocker and those not using beta-blockers. To this end, the average of the pre-treatment indices of both dialysis modalities was calculated and compared between beta-blocker users and non-users with a Mann-Whitney ranked sum test. Analyses were performed with SPSS version 22.0 and GraphPad Prism version 5.0. *P*-values of < 0.05 (two-tailed) were considered statistically significant.

## Results

### Patients

**Fig. 1:**
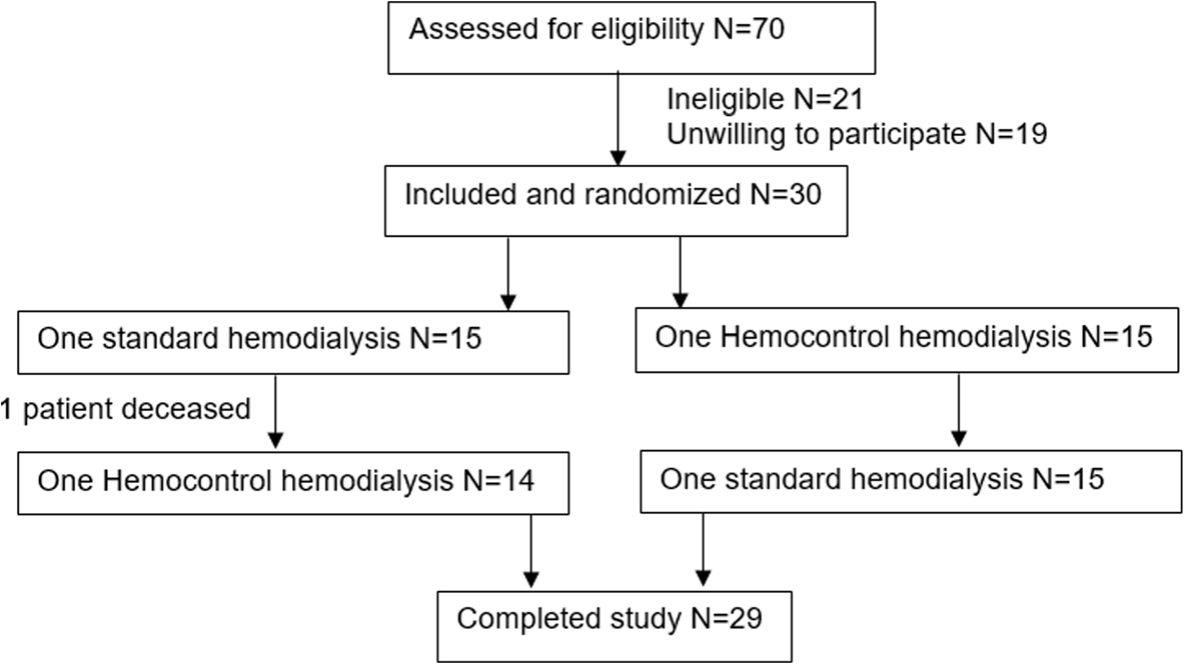
Flow diagram of participants

Twenty-nine patients completed the study protocol (Fig. 1). The mean (± SD) age was 63 ± 17 years and 21 (72%) of the participants were male. Eight patients (28%) had diabetes mellitus and 26 patients (90%) had hypertension. Median dialysis vintage was 25.0 [IQR 14.5–48.5] months. Fifteen patients (52%) had residual renal function, with a median residual diuresis of 250 ml/day [IQR 0–750]. The causes of renal failure were hypertension (*n* = 8), diabetes mellitus (*n* = 3), autosomal polycystic kidney disease (*n* = 2), glomerulonephritis (*n* = 4), tubulo-intersitial nephritis (*n* = 1), urologic cause (*n* = 2), atherosclerosis (*n* = 1), renal clear cell carcinoma (*n* = 1), horseshoe kidney (n = 1); in six patients the cause of renal failure was unknown. Cardiovascular medication was used by 27 patients: angiotensin converting enzyme inhibitor *n* = 3 (10%), angiotensin receptor blocker n = 3 (10%), beta-blocker *n* = 20 (69%), calcium channel blocker *n* = 7 (24%), diuretic *n* = 4 (14%), statin *n* = 9 (31%), aspirin *n* = 20 (69%), doxazosine *n* = 3 (10%) and nitrate *n* = 2 (7%).

Pre- and postdialysis weight, total ultrafiltration volume, and relative blood volume and ΔBV/UF ratio at the end of treatment were similar for the two treatment modalities. Predialysis weight was 82.1 kg (±16.2) and 82.3 kg (±16.3) (*p* = 0.28) and postdialysis weight was 80.0 kg (±16.1) and 80.1 kg (±16.3) (*p* = 0.50) for standard hemodialysis and Hemocontrol dialysis, respectively.

### Plasma sodium and osmolality

**Fig. 2:**
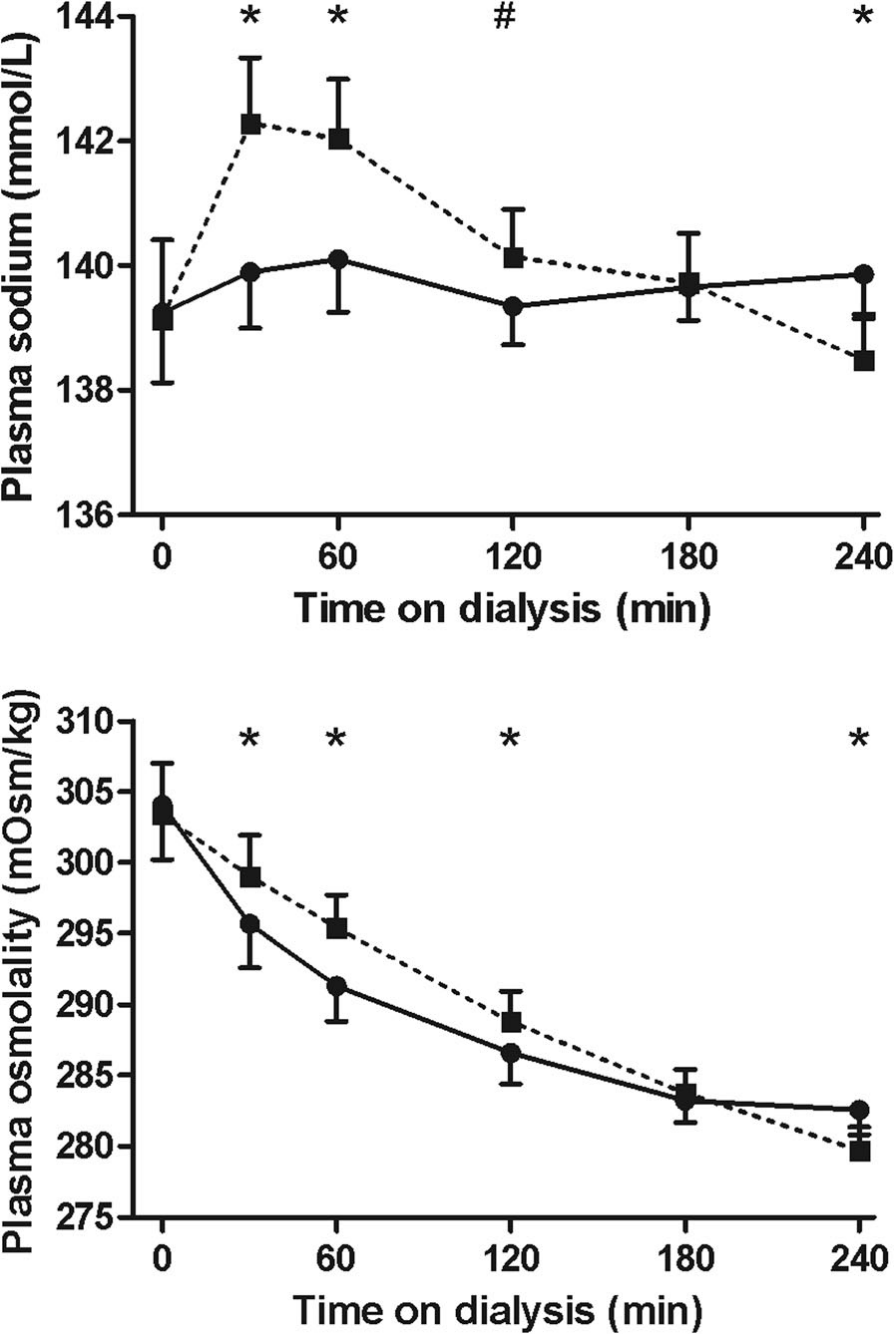
Plasma sodium and osmolality during hemodialysis. Plasma sodium (upper panel) and plasma osmolality (lower panel) during hemodialysis (mean ± 95% CI). Legend: standard hemodialysis; Hemocontrol hemodialysis. # Denotes *p* < 0.05 for the difference between standard hemodialysis and Hemocontrol hemodialysis. *Denotes *p* < 0.01 for the difference between standard hemodialysis and Hemocontrol hemodialysis

**Fig. 3:**
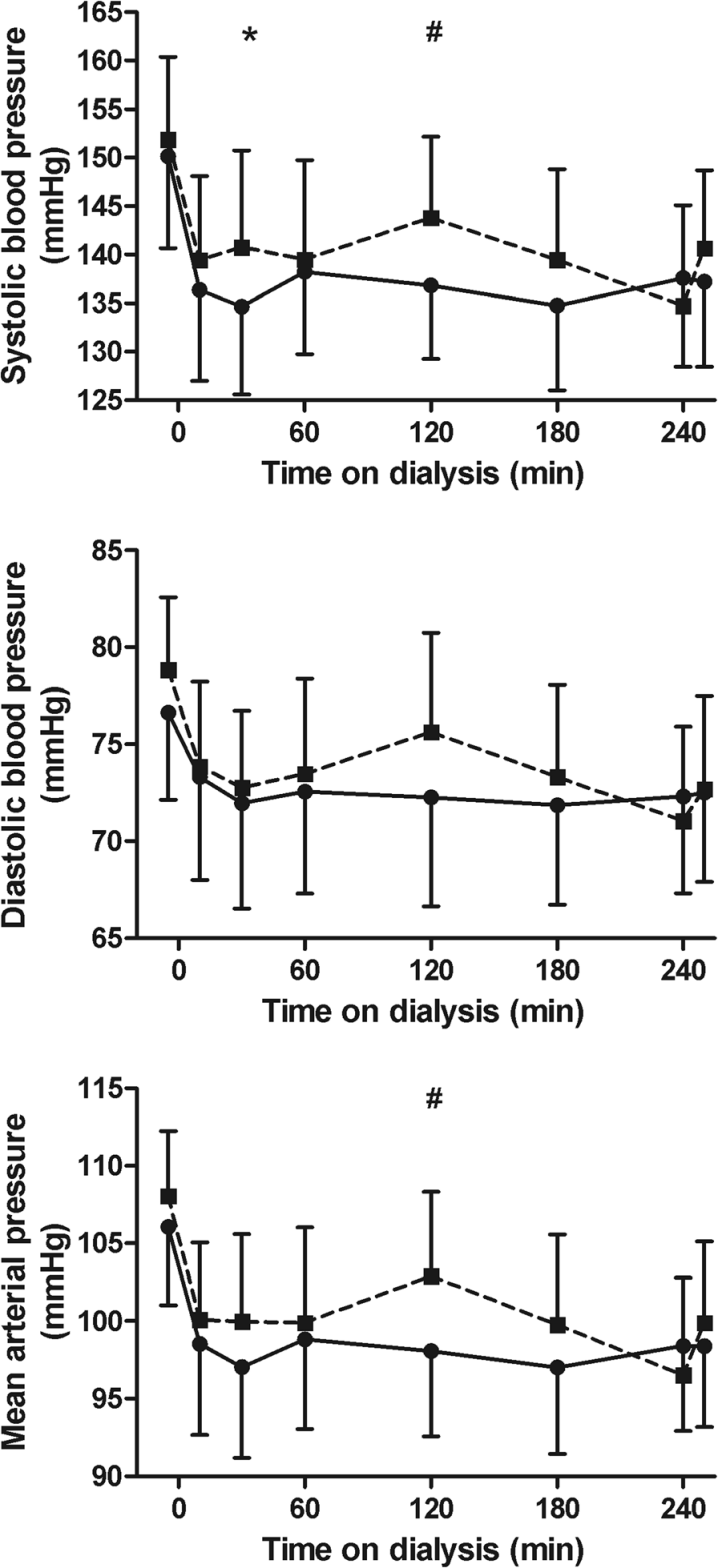
Systolic and diastolic blood pressure, mean arterial pressure and heart rate during hemodialysis. Systolic blood pressure (upper left panel), diastolic blood pressure (upper right panel) and mean arterial pressure (lower left panel) during hemodialysis (mean ± 95% CI). Legend: standard hemodialysis; Hemocontrol hemodialysis. # Denotes *p* < 0.05 for the difference between standard hemodialysis and Hemocontrol hemodialysis. * Denotes *p* < 0.01 for the difference between standard hemodialysis and Hemocontrol hemodialysis

Plasma sodium and plasma osmolality differed between the two treatment modalities and were significantly higher during the first half of Hemocontrol dialysis (Figs. 2 and 3).

### Hemodynamic parameters

**Fig. 4:**
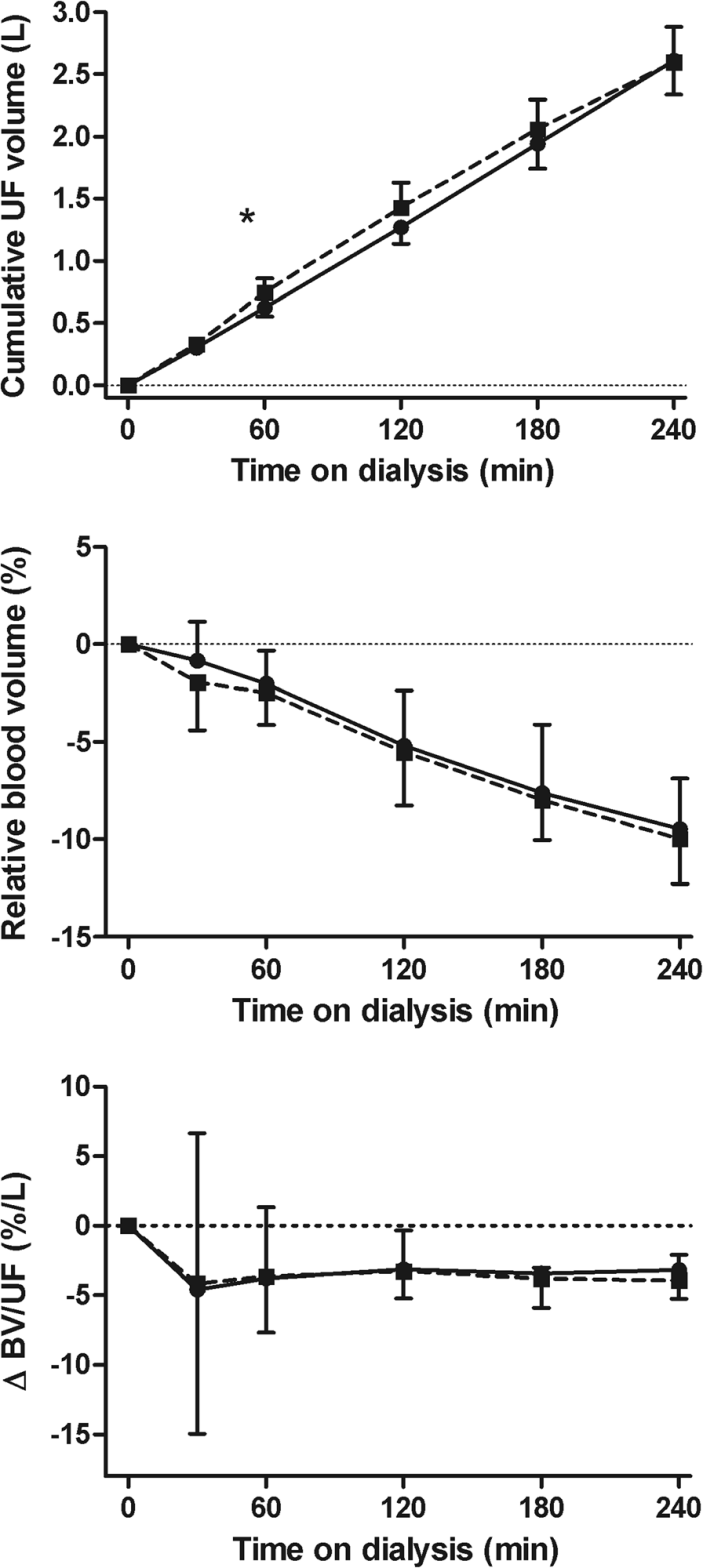
Ultrafiltration volume, ΔRBV and ΔBV/UF ratio during hemodialysis. Ultrafiltration volume (upper panel), ΔRBV (middle panel) (mean ± 95% CI) and ΔRBV/UF ratio (median and interquartile range) during hemodialysis. Abbreviations: BV: blood volume; UF: ultrafiltration volume. Legend: standard hemodialysis; Hemocontrol hemodialysis. * Denotes *p* < 0.01 for the difference between standard hemodialysis and Hemocontrol hemodialysis

The course of systolic blood pressure and MAP differed significantly between Hemocontrol hemodialysis and standard hemodialysis and this difference was most marked at 120 min on dialysis, with a significantly higher MAP with Hemocontrol (Fig. 4). The course of heart rate did not differ between the two treatments throughout the dialysis session (*p* = 0.64) (77 and 76 bpm predialysis and 76 and 75 bpm postdialysis for standard and Hemocontrol dialysis, respectively). In line with the concept of Hemocontrol, the higher ultrafiltration rate in the beginning of dialysis resulted in a slightly but significantly higher initial cumulative ultrafiltration volume during treatment (*p* < 0.001) (Fig. 4). At the end of treatment, the total ultrafiltration volume was similar for both treatment modalities. The course of RBV was also similar for the two treatments (*p* = 0.97) and decreased to − 9.5 and − 10% at the end of standard hemodialysis and Hemocontrol dialysis, respectively (Fig. 4). The intradialytic course of ΔRBV/UF did not differ between the two treatment modalities (*p* = 0.95), indicating no difference in plasma refill rate between the two treatments (Fig. 4).

### Intradialytic course of blood pressure regulators

#### Plasma vasopressin and copeptin

**Fig. 5:**
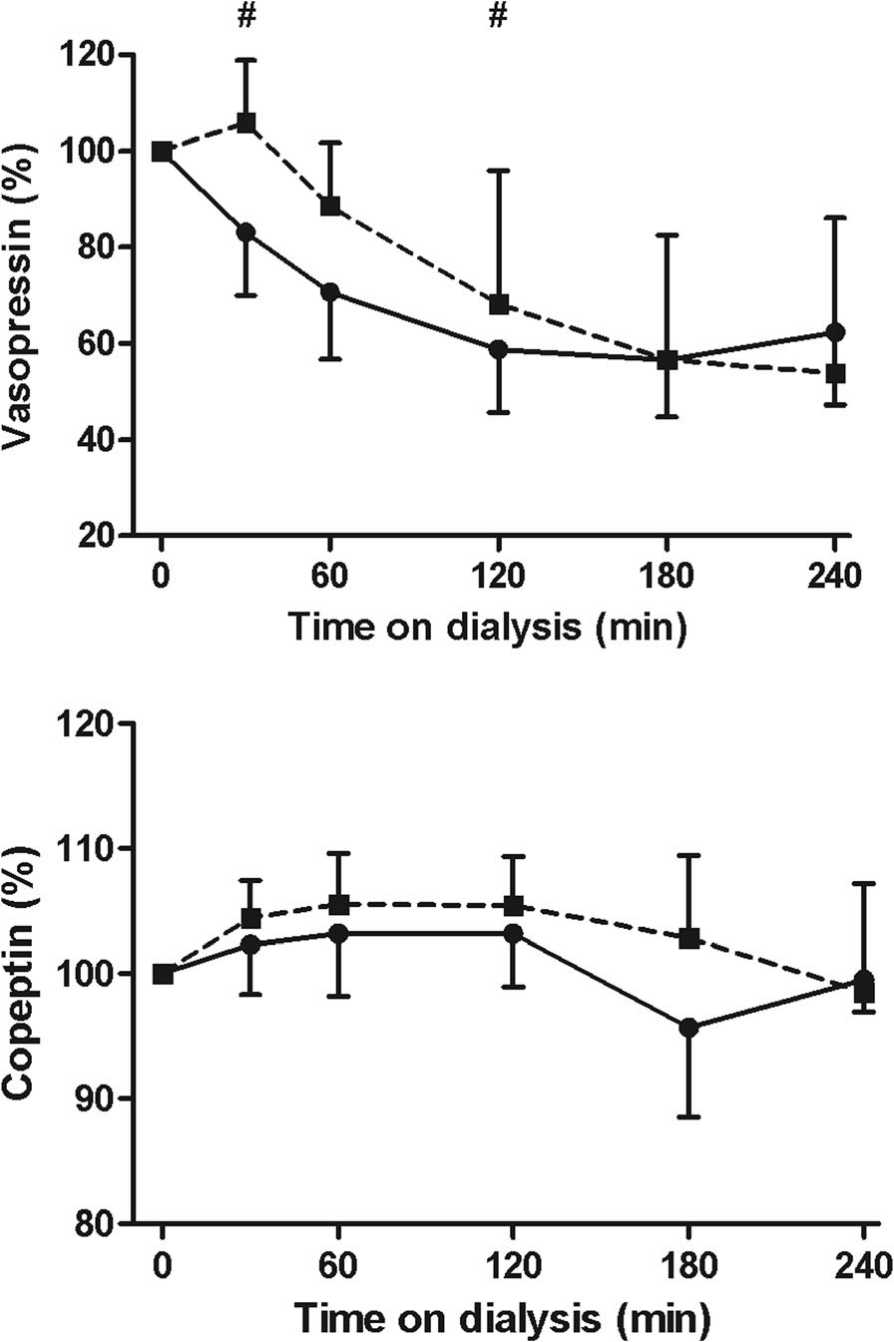
Change in plasma vasopressin and copeptin during hemodialysis. Median and interquartile range. Legend: standard hemodialysis; Hemocontrol hemodialysis. # Denotes *p* < 0.05 for the difference between standard hemodialysis and Hemocontrol hemodialysis

The overall course of plasma vasopressin levels differed between the two dialysis treatments. Vasopressin levels were significantly higher at 30 and 120 min on Hemocontrol dialysis (Fig. 5). In contrast, the course of plasma copeptin levels did not differ between the two treatment modalities (*p* = 0.23) (Fig. 5).

#### Indices of sympathetic activity

**Fig. 6:**
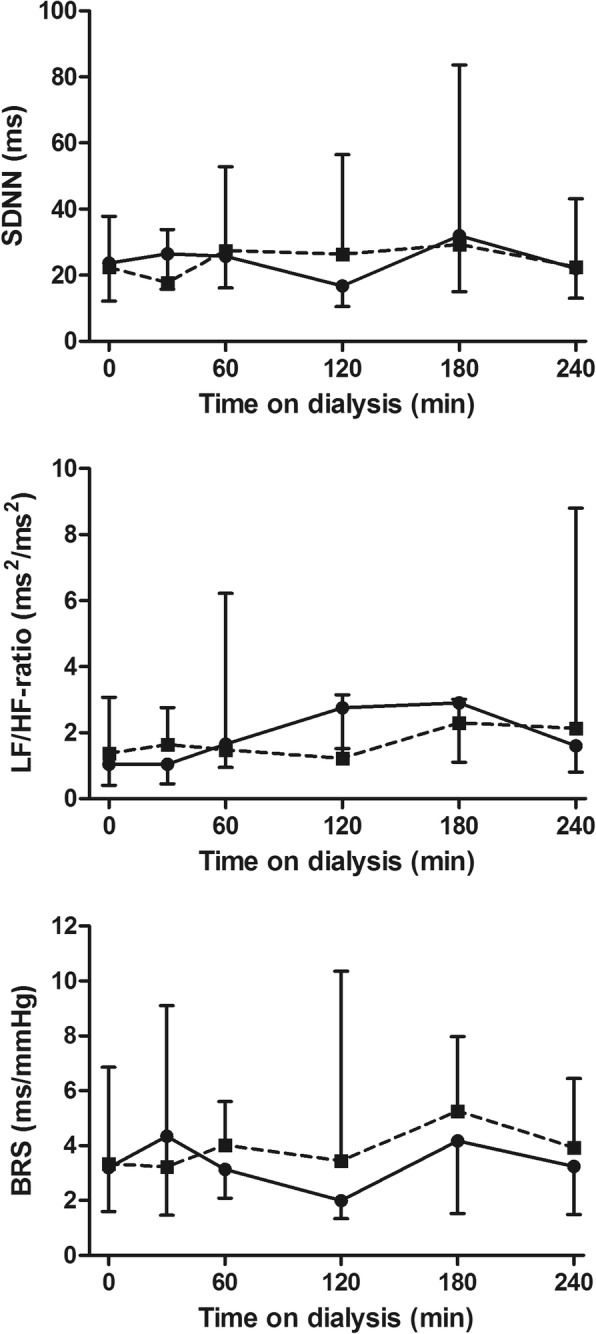
SDNN, LF/HF-ratio and BRS during hemodialysis. SDNN (upper panel; *N* = 11), LF/HF-ratio (middle panel; *N* = 11) and BRS (lower panel; *N* = 9) during hemodialysis (median and interquartile range). Abbreviations: SDNN: standard deviation of interbeat intervals (NN intervals); LF: low frequency; HF: high frequency; BRS: baroreflex sensitivity. Legend: standard hemodialysis; Hemocontrol hemodialysis

Complete (i.e. at all 6 time points and during both treatments) measurement of heart rate variability and BRS was technically possible in 11 (38%) and 9 (31%) of the 29 patients, respectively. There were no differences between standard hemodialysis and Hemocontrol hemodialysis in the course of SDNN (*p* = 0.26), LF/HF ratio (*p* = 0.26) or BRS (*p* = 0.24) (Fig. 6).

**Fig. 7:**
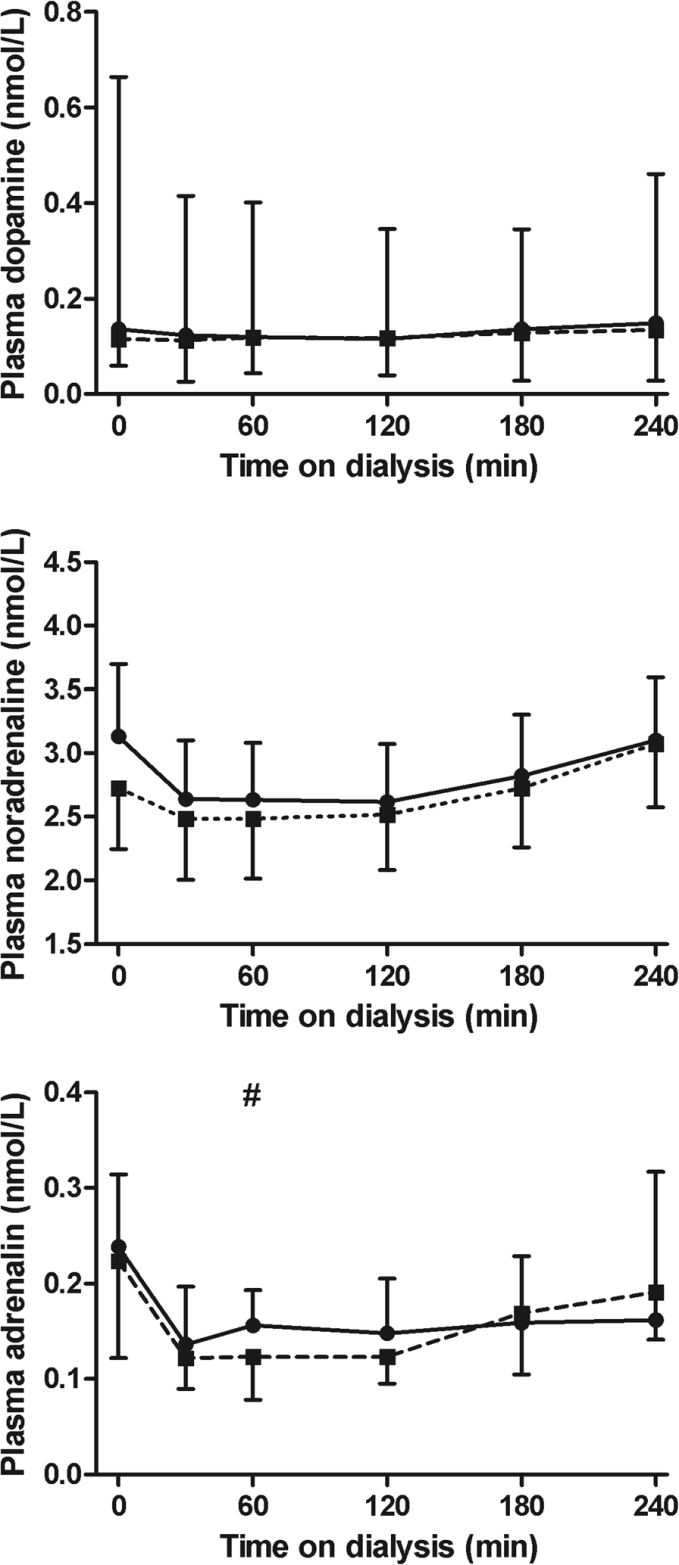
Plasma catecholamines during hemodialysis. Plasma dopamine (upper panel, median and interquartile range), noradrenalin (middle panel, mean ± 95% CI) and adrenalin (lower panel, median and interquartile range) during hemodialysis. Legend: standard hemodialysis; Hemocontrol hemodialysis. # Denotes *p* < 0.05 for the difference between standard hemodialysis and Hemocontrol hemodialysis

The course of plasma dopamine and noradrenalin levels did not differ between the two treatment modalities (*p* = 0.88 and *p* = 0.59, respectively). Plasma adrenalin levels were higher at 60 min on standard hemodialysis compared with Hemocontrol dialysis (median 0.04 nmol/L, IQR -0.004–0.060; *p* = 0.02) (Fig. 7).

#### Indices of endothelial function

**Fig. 8:**
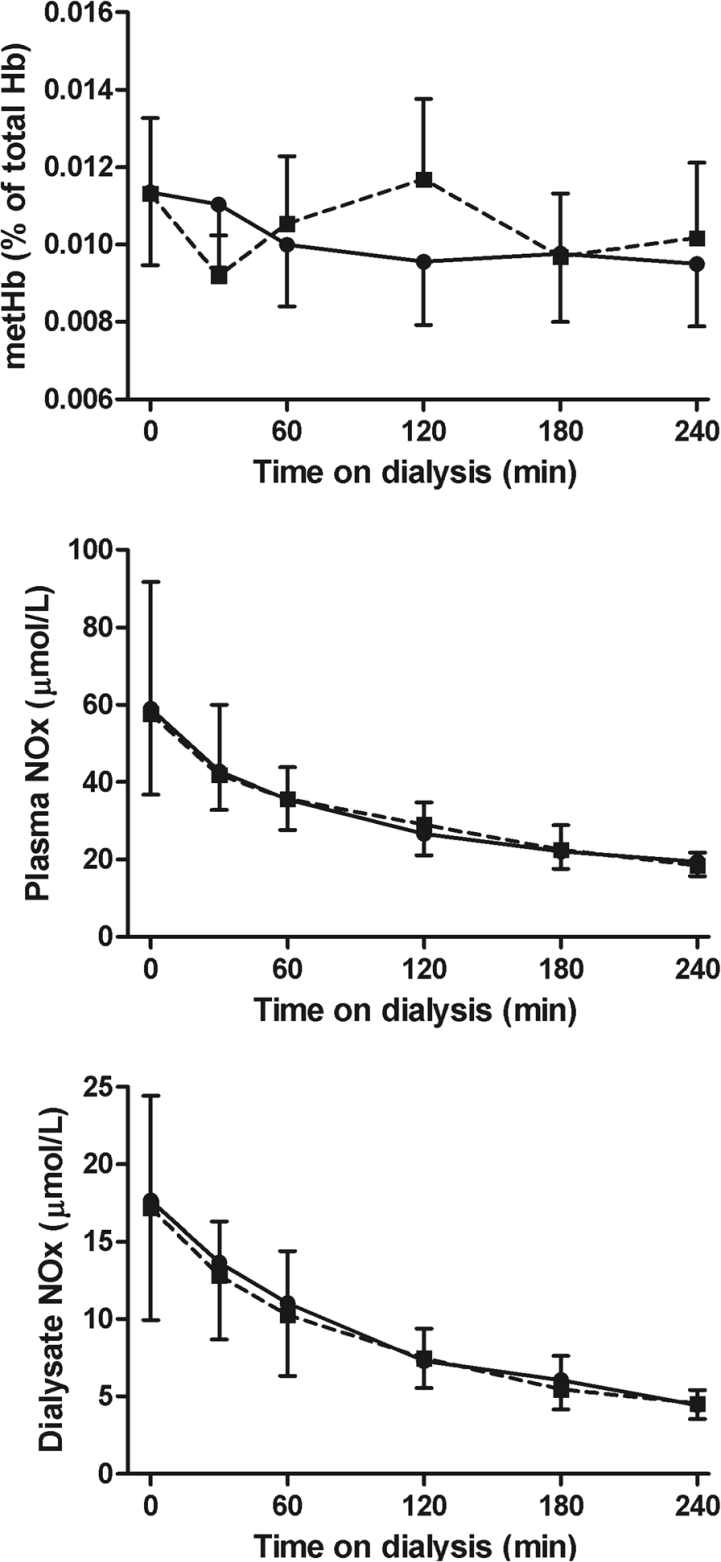
Methemoglobin and plasma NOx levels during hemodialysis. Methemoglobin (mean ± 95% CI) and plasma NOx (median and interquartile range) during hemodialysis. Abbreviations: NOx: nitrite and nitrate. Legend: standard hemodialysis; Hemocontrol hemodialysis

**Fig. 9:**
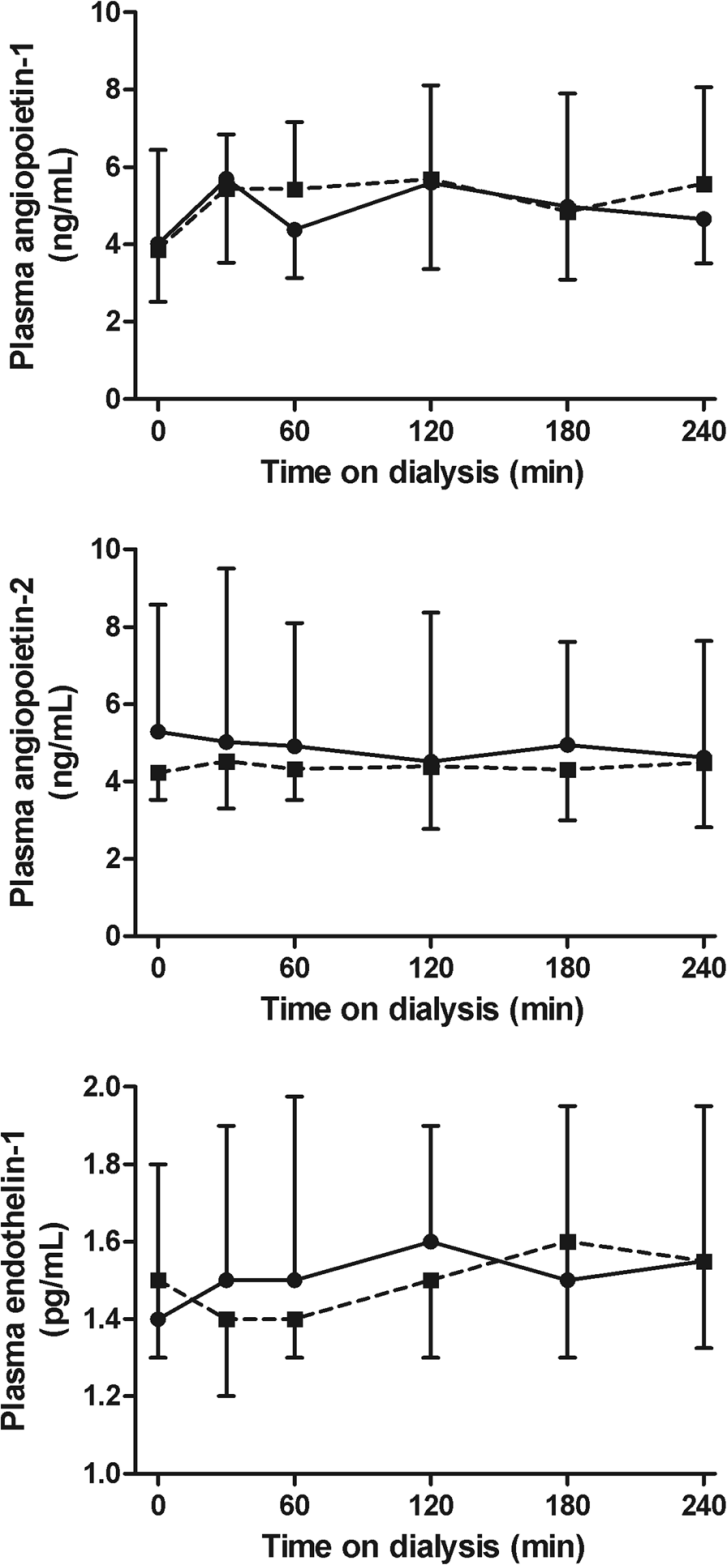
Plasma Ang1, Ang2 and endothelin-1 levels during hemodialysis. Plasma Ang1 (upper panel), Ang2 (middle panel) and endothelin-1 (lower panel) during hemodialysis (median and interquartile range). Abbreviations: Ang1: angiopoietin-1; Ang2: angiopoietin-2. Legend: standard hemodialysis; Hemocontrol hemodialysis

The courses of all measured endothelial function parameters were similar for the two treatment modalities. There was no significant difference in metHb (*p* = 0.07) or plasma NOx levels (*p* = 0.51) (Fig. 8) between standard hemodialysis and Hemocontrol dialysis. Also the course of nitrite and nitrate separately did not differ between the two treatment modalities (*p* = 0.28 and *p* = 0.50, respectively). Intradialytic courses of plasma endothelin-1 (*p* = 0.07) and Ang1 and Ang2 levels (*p* = 0.25 and *p* = 0.34, respectively) did not differ significantly between treatments (Fig. 9).

### Association between vasopressin and blood pressure

To test whether the intradialytic course of plasma vasopressin levels was associated with the intradialytic course of MAP for the two treatments, a triple interaction term (time×treatment×vasopressin) was added to the model. This interaction term was significant (*p* < 0.001). When the two treatment modalities were analyzed separately, the interaction term (time×vasopressin) was not significant during standard hemodialysis dialysis (*p* = 0.10), whereas it was significant during Hemocontrol dialysis (*p* = 0.02).

### Sensitivity analyses

When the GEE analyses of the intradialytic course of the study parameters and the association between vasopressin and MAP were adjusted for age, gender, diabetes mellitus, dialysis vintage, and residual renal function, the results were similar. The results of the intradialytic courses of systolic blood pressure and MAP remained similar when the first intradialytic measurement of blood pressure was included in the analyses instead of the predialysis measurement. When endothelin-1, Ang-1 and Ang2, vasopressin, and copeptin were not corrected for hemoconcentration, comparable results were obtained.

Patients using a beta-blocker had, compared with patients without beta-blockers, a significantly lower SDNN (17.2 ms [IQR 14.7–28.8]; *N* = 10 versus 40.6 ms [33.1–75.3]; *N* = 8; *p* = 0.002) and BRS (2.5 ms/mmHg [1.7–2.7]; N = 8 versus 7.5 ms/mmHg [4.1–9.9]; *N* = 6; *p* = 0.003) predialysis. The difference between predialysis LF/HF-ratio in patients with and without a beta-blocker was not significant (2.4 ms^2^/ms^2^ [0.9–4.3]; N = 10 versus 0.7 ms^2^/ms^2^ [0.5–1.8]; N = 8; *p* = 0.07, respectively).

## Discussion

In this study it was investigated whether Hemocontrol dialysis, characterized by an initial and transient increase in plasma sodium levels, affected the intradialytic course of several blood pressure regulators in comparison with standard hemodialysis. It was observed that the course of various markers of endothelial function and sympathetic activity were similar during both hemodialysis modalities. The only difference between the two treatments was a slightly but significantly higher initial vasopressin level during Hemocontrol dialysis.

Several studies have showed improved intradialytic hemodynamic stability with Hemocontrol dialysis [23, 37–39]. In the present study, also an overall higher intradialytic blood pressure during dialysis with Hemocontrol compared with standard hemodialysis was observed. The general believe is that higher dialysate sodium levels increase plasma refilling from the interstitial tissue [4, 7, 9, 40] and that blood volume is better preserved with Hemocontrol in comparison with standard hemodialysis. However, no better blood volume preservation with Hemocontrol was observed previously [38, 39]. Thus, the improved hemodynamic stability with Hemocontrol is not easily explained by an effect on blood volume and presumably other mechanisms play a role. From in-vitro studies it was suggested that an increase in sodium, to the same extent as observed in our study, affected endothelial cells and caused a down-regulation of NO [13, 17, 41]. Less NO production could ameliorate the intradialytic hemodynamic stability. In-vivo studies, however, showed less consistent effects of sodium on NO production. In some studies, plasma NOx levels changed opposite to the daily amount of salt intake in humans, suggestive of an effect of sodium on endothelial function [42–44], whereas other studies did not find such an association [45, 46]. Very few studies in hemodialysis patients have been conducted. To our knowledge, one other group studied the effect of sodium on endothelial function during hemodialysis and did not find a difference in intradialytic plasma levels of nitrite and endothelin-1 between hemodialysis with low and high dialysate sodium levels [47], which is in line with our findings.

In the present study, also no differences in various indices of sympathetic activity were observed between standard hemodialysis and Hemocontrol hemodialysis. The pre-treatment indices of autonomic function were low, with BRS slightly above 3 ms/mmHg and SDNN as a measure of overall HRV around 22 ms predialysis. The presence of a low HRV in hemodialysis patients is in line with previous findings [48]. Our findings indicate that autonomic dysfunction is prevalent in this population and that the patients were unable to modulate sympathetic activity during hemodialysis, even in the presence of initially higher plasma sodium levels and ultrafiltration rate. As far as we know, three other groups studied the effect of dialysate sodium on autonomic activity and also did not find a stimulating effect of higher sodium levels on sympathetic activity [48–50].

We cannot completely exclude that the higher initial ultrafiltration volume in Hemocontrol dialysis provided an additional stimulus besides osmolality for vasopressin release compared with standard hemodialysis. However, the course of blood volume and ΔRBV/UF were similar for the two treatment modalities. Our results do not support the concept that the improved intradialytic hemodynamic stability with Hemocontrol is mediated by an enhanced plasma refill rate since we did not find a higher plasma refill with Hemocontrol in response to the initially higher plasma sodium levels. This is in contrast with the general believe that a higher plasma sodium concentration enhances plasma refill rate. Although several groups concluded that a temporary increase in dialysate sodium concentration led to an enhanced plasma refill rate [38, 51, 52], other studies also did not find a significant increase in plasma refill rate when a higher dialysate conductivity was used [6, 10, 53]. The difference between studies could be explained by different methods used to estimate the plasma refill rate. These methods, including ours, might not be sensitive enough to accurately reflect refill. Additionally, studies may have differed in the achieved intradialytic plasma sodium concentrations, which could have led to differences in osmotic force driving plasma refill.

A higher dialysate sodium concentration may also improve intradialytic hemodynamic stability through a stimulating effect on vasopressin release as has been suggested before [54]. Indeed, previous findings indicate that intradialytic administration of hypertonic saline increased blood pressure via vasopressin release rather than via expansion of the intravascular volume [55, 56]. In the present study, plasma vasopressin levels were initially higher with Hemocontrol dialysis. Although the difference in plasma vasopressin levels between the two treatment modalities was small, the observed intradialytic pattern was similar to the pattern observed in our previous study in a different patient group [10]. Removal of vasopressin by dialysis [57], may have precluded a (greater) rise in vasopressin levels during both treatments.

Unlike vasopressin, the course of plasma copeptin levels did not differ significantly between the two treatment modalities. Copeptin is increasingly used as a surrogate marker for vasopressin levels because it is easier to measure and assumed to be more stable ex vivo [24, 58–60]. Copeptin levels were increased at the end of both dialysis treatments compared to pre-treatment. Thus, we could speculate that release of copeptin was present during both dialysis treatments. It is generally assumed that vasopressin and copeptin are secreted together from the pituitary gland upon stimuli [24, 60, 61], thus differences in intradialytic clearance of 3.5 ml/min versus 61.5 ml/min for copeptin and vasopressin, respectively [57], and potentially the half-life of copeptin and vasopressin might explain the divergent courses of these peptides during conventional hemodialysis.

This study has a number of limitations that need to be acknowledged. First, since this was a short-term study, results may not be representative of long-term application of dialysate sodium modification. Second, we did not specifically include hypotension-prone patients, implicating that our findings may not be generalizable for this specific patient group. Third, the measurement of HRV has limitations and the recordings were only technically useful, i.e. with limited signal irregularities requiring RR peak interpolation, in about one third of the patients. However, catecholamines were measured in all patients and the intradialytic courses of catecholamine levels were similar to (dopamine and noradrenalin) or even lower (adrenalin) in Hemocontrol hemodialysis compared with standard hemodialysis. Strengths of the present study are the cross-over design and the repeated measurement of multiple indices of endothelial function and sympathetic activity during dialysis. Furthermore, changes in blood volume were derived from laboratory-measured hemoglobin levels instead of Hemoscan since this technique may be affected by changes in plasma osmolality [62, 63]

## Conclusions

In conclusion, a transient rise in plasma sodium levels during Hemocontrol hemodialysis did not result in a different course of various indices of endothelial function and sympathetic activity compared with standard hemodialysis, but coincided with significantly higher plasma levels of vasopressin as well as a higher mean arterial pressure. Our study suggests that the beneficial effect of an increase in plasma sodium concentration on intradialytic hemodynamic stability might be mediated by vasopressin.

## Abbreviations

∆RBV: changes in relative blood volume
∆RBV/UF: change in RBV normalized for ultrafiltration volume
Ang1 and Ang2: angiopoietin-1 and 2
BRS: baroreflex sensitivity
GEE: generalized estimating eqs.
HF: high frequency
HRV: heart rate variability
LF: low frequency
MAP: mean arterial pressure
metHb: methemoglobin
NN intervals: interbeat intervals
NO: nitric oxide
NOx: nitrite and nitrate
SDNN: standard deviation of the interbeat intervals
